# Protein Intakes and Serum Albumin Levels in a Japanese General Population: NIPPON DATA90

**DOI:** 10.2188/jea.JE20090221

**Published:** 2010-05-05

**Authors:** Makoto Watanabe, Aya Higashiyama, Yoshihiro Kokubo, Yuu Ono, Akira Okayama, Tomonori Okamura

**Affiliations:** 1 Department of Preventive Cardiology, National Cardiovascular Center, Osaka, Japan; 2 The First Institute for Health Promotion and Health Care, Japan Anti-tuberculosis Association, Tokyo, Japan

**Keywords:** serum albumin, protein intake, cross-sectional study

## Abstract

**Background:**

It is well-known that albumin is synthesized in the liver; serum albumin is a major component of serum proteins. However, it has not been well elucidated how dietary protein intakes are associated with serum albumin levels in general populations without extreme malnutrition. We cross-sectionally investigated in the representative Japanese the association between dietary protein intake and serum albumin levels.

**Methods:**

A total of 7715 subjects (3220 men and 4495 women, aged 30 years or more) with measurement of serum albumin who participated in both the National Survey on Circulatory Disorders in 1990 and the National Nutrition Survey in 1990 were analyzed in the present analysis. Multiple-adjustments were performed with linear regression models to estimate the association between serum albumin levels and animal or vegetable protein intake adjusting for age and body mass index.

**Results:**

The very weak positive association between animal protein and serum albumin levels was observed. On the other hand, there was no clear association observed between vegetable protein and serum albumin levels. Regardless of sex and models, age was inversely associated with serum albumin levels with statistically significance, and standardized coefficients of age were considerably larger in both sexes than other variables. Adjustment for body mass index hardly altered the coefficients of animal or vegetable protein intake, but adjustment for total cholesterol clearly attenuated the relationship between animal protein intake and serum albumin levels.

**Conclusions:**

Present analysis indicated the possibility that animal protein intake was related with serum albumin levels, while vegetable protein intake was not related.

## INTRODUCTION

It is well-known that albumin is synthesized in the liver. Serum albumin is a major component of serum protein, which sustains osmotic pressure or transports many kinds of substances or hormones to organs. Serum albumin level can be lowered by decrease in synthesis (cirrhosis or some inflammatory diseases), leakage outside the body (nephrotic syndrome, severe burns or protein-losing enteropathy), or malnutrition.

Several prospective studies have reported that low albuminemia was associated with risks of mortality or cardiovascular diseases^1^^–^^7^; however, the mechanism has not been clear enough. As for serum albumin levels, there is one simple question if dietary protein intake is associated with serum albumin levels in a general population without extreme malnutrition. Solution of this question might be one of clues to solve the mechanism between serum albumin and adverse health. Accordingly, we cross-sectionally investigated in the representative Japanese the association between dietary protein intakes and serum albumin levels.

## METHODS

### Study subjects

The National Survey of Circulatory Disorders, Japan (NSCDJ) and the National Nutrition Survey, Japan (NNSJ) are periodically conducted for same participants (NSCDJ: every 10 years, NNSJ: every year) in the randomly sampled subjects from all the Japanese.^8^^,^^9^

In 1990, both these national surveys were conducted to the 10 956 same subjects who lived in the randomly selected 300 areas from Japan. Among them, 8926 subjects participated in the surveys (participation rate: 81%). Finally, a total of 7715 subjects (3220 men and 4495 women, aged 30 years or more) with measurement of serum albumin and nutrient intake data remained for the present analysis.

The cohort study of NSCDJ in 1990 (NSCDJ90) is referred to as NIPPON DATA 90 (the National Integrated Project for Prospective Observation of Non-communicable Disease And its Trends in the Aged). Details of NIPPON DATA 90 were previously described elsewhere.^10^^–^^12^

### Data collection in NSCDJ90

Public health nurses obtained information on cigarette smoking, alcohol drinking, or medication for hypertension.^8^ Blood pressure was measured using a standard mercury sphygmomanometer on the right arm of seated participants after a sufficient period of rest. Non-fasting blood samples were obtained and the serum was separated and centrifuged soon after blood coagulation. Plasma samples were also obtained in a siliconized tube containing sodium fluoride. These samples were shipped to a unique laboratory (SRL, Tokyo, Japan) for blood measurements. The serum albumin was analyzed with the bromcresol green method.

### Data collection in NNSJ in 1990

Since nutrient intakes were estimated at the NNSJ in 1990 (NNSJ90) by each household with food weighing method,^9^ those of each household member were estimated by proportionally dividing household intake data of NNSJ90, based on average intakes by sex and age groups calculated from NNSJ in 1995. The details of the estimation for individual nutrient intakes are described in another paper.^13^ Intakes of total protein, and animal or vegetable protein were calculated as g per day and caloric densities in g/1000 kcal.

### Statistical analysis

Serum albumin level was divided into three groups by its tertile to compare the characteristics. One-way analysis of variance for continuous variables and chi-square test for categorical variables were used for comparison among groups. Multiple-adjustments were performed with linear regression models to estimate association between serum albumin levels and animal or vegetable protein intake; Model 1 including age, animal and vegetable protein intake, Model 2 including variables in model 1 plus body mass index (BMI), Model 3 including variables in model 2 plus total cholesterol. In addition, as sensitivity analyses, current cigarette smoking and current alcohol drinking were added to the model 1 as dichotomized variables, and daily animal or vegetable protein intake per energy intake of 1000 kcal was entered into the model 2, instead of animal or vegetable protein intake as g per day. Adjusted coefficient of determination (adjusted *R*^2^) was also calculated. All *P*-values were 2-tailed and the significance level was *P* < 0.05. The statistical package SPSS 12.0J for Windows (SPSS, Tokyo, Japan) performed these analyses.

## RESULTS

Average protein intake (total, animal and vegetable), BMI, and serum albumin levels were shown by age groups and sex (Table 1). All of these variables differed significantly among age groups, and serum albumin levels decreased with age, especially more clearly in men. Regardless of sex, total, animal and vegetable protein intake is lowest in 70 years or more. BMI is higher in 40–49 years in men and in 50–69 years in women than other age groups. Prevalence of low albuminemia of 38 g/L or less were very low in both sexes; men: 2.2% (*n* = 70), women: 1.7% (*n* = 77) (table not shown).

**Table 1.** Averages in protein intakes (total, animal, vegetable), serum albumin levels and body mass index by age groups and sex, 1990, Japan

Men		Protain intakes (g/day)			Serum albumin (g/L)	BMI (kg/m^2^)
	
Age	*n*	Total	Animal	Vegetable		
30–39	606	88.7 (18.1)	45.8 (13.4)	42.9 (8.7)	46.9 (2.5)	22.9 (3.0)
40–49	767	93.1 (18.1)	49.2 (13.8)	43.8 (8.5)	45.8 (2.7)	23.5 (2.9)
50–59	737	96.8 (20.6)	50.4 (15.5)	46.4 (9.5)	44.6 (2.5)	23.3 (2.8)
60–69	662	87.2 (18.2)	43.5 (13.4)	43.9 (9.1)	43.4 (2.7)	22.6 (3.1)
70–	448	78.1 (17.7)	38.4 (12.9)	39.8 (8.9)	42.2 (2.7)	22.0 (3.1)
*P* value		<0.001	<0.001	<0.001	<0.001	<0.001



Women		Protain intakes (g/day)			Serum albumin (g/L)	BMI (kg/m^2^)
	
Age	*n*	Total	Animal	Vegetable

30–39	976	71.9 (13.0)	37.0 (9.9)	34.9 (6.3)	44.9 (2.6)	21.8 (3.0)
40–49	1097	78.2 (15.3)	41.1 (11.4)	37.1 (7.2)	44.4 (2.4)	22.8 (3.1)
50–59	967	78.1 (16.8)	39.7 (12.3)	38.5 (8.1)	44.4 (2.5)	23.4 (3.2)
60–69	847	72.6 (16.3)	35.6 (11.5)	37.1 (8.2)	43.9 (2.5)	23.4 (3.5)
70–	608	64.3 (14.2)	31.0 (10.3)	33.5 (7.1)	42.5 (2.7)	22.8 (3.4)
*P* value		<0.001	<0.001	<0.001	<0.001	<0.001

*P* values were calculated between age groups with one-way anova.

Parentheisis means standard deviation.

BMI means body mass index.

Average protein intake (total, animal and vegetable) per energy intake of 1000 kcal were shown (Table 2). In both sexes, total protein intake per energy intake was highest in 50–59 years, and vegetable protein intake per energy intake increased with age and was highest in 70 years or more. Animal protein intake per energy intake was higher in 40–59 years than other age groups.

**Table 2.** Averages in protein intakes (total, animal, vegetable) per energy intake of 1000 kcal by age groups and sex, 1990, Japan

Men		Protain intakes(g/1000 kcal)		
	
Age	*n*	Total	Animal	Vegetable
30–39	606	37.2 (4.3)	19.3 (4.5)	18.0 (2.0)
40–49	767	38.8 (4.6)	20.5 (4.7)	18.2 (2.2)
50–59	737	39.7 (5.0)	20.6 (5.0)	19.0 (2.3)
60–69	662	38.9 (4.8)	19.4 (4.9)	19.6 (2.5)
70–	448	39.3 (5.1)	19.3 (5.4)	20.1 (2.7)
*P* value		<0.001	<0.001	<0.001



Women		Protain intakes(g/1000 kcal)		
	
Age	*n*	Total	Animal	Vegetable

30–39	976	38.3 (4.2)	19.7 (4.4)	18.6 (2.1)
40–49	1097	39.9 (4.8)	21.0 (4.8)	19.0 (2.3)
50–59	967	40.7 (5.1)	20.7 (5.2)	20.1 (2.4)
60–69	847	40.3 (5.2)	19.7 (5.2)	20.6 (2.6)
70–	608	39.9 (5.3)	19.2 (5.3)	20.8 (2.6)
*P* value		<0.001	<0.001	<0.001

*P* values were calculated between age groups with one-way anova.

Parentheisis means standard deviation.

The characteristics by tertile of serum albumin levels were shown (Table 3). In both sexes, averages in age, total energy intake, total cholesterol, total and animal protein intakes per day, and proportion of current alcohol drinker differed significantly among categories of serum albumin levels. Animal or vegetable protein intake per energy intake of 1000 kcal also differed significantly among categories. No significant difference was observed in vegetable protein intake regardless of sex. Average BMI differed significantly only in men, and increased with serum albumin levels. In addition, average animal and vegetable protein intake were lower in low-albuminemia (albumin level of 38 g/L or less) than normal-albuminemia in both sexes, although difference for animal protein in women was not significant (table not shown).

**Table 3.** Comparison of characteristics by tertiles of serum albumin levels and sex, 1990, Japan

Men	Serum albumin (g/L)			*P*-value
	
	29–43	44–46	47–56	
	
*n*	1080	1231	909	
Age (years)	62.1 (12.2)	51.9 (12.0)	44.7 (10.8)	<0.001
BMI (kg/m^2^)	22.3 (2.9)	23.2 (3.1)	23.4 (2.9)	<0.001
Total cholesterol (mg/dL)	188 (35)	201 (35)	209 (37)	<0.001

Total energy intake (kcal/day)	2254 (484)	2351 (465)	2372 (413)	<0.001
Protein (g/day)	87.3 (19.7)	91.0 (20.0)	91.3 (18.5)	<0.001
Animal (g/day)	44.0 (14.1)	47.0 (14.8)	47.6 (14.3)	<0.001
Vegetable (g/day)	43.4 (9.6)	43.9 (9.2)	43.7 (8.4)	0.41
Protein (g/1000 kcal)	38.9 (4.6)	38.9 (5.0)	38.6 (4.8)	0.31
Animal (g/1000 kcal)	19.6 (4.8)	20.1 (5.0)	20.1 (4.9)	0.02
Vegetable (g/1000 kcal)	19.4 (2.5)	18.8 (2.4)	18.5 (2.3)	<0.001

Current alcohol drinker (%)	53.1	58.2	63.5	<0.001
Current cigarette smoker (%)	54.0	55.2	57.4	0.30



Women	Serum albumin (g/L)			*P*-value
	
	31–43	44–45	46–54	
	
*n*	1768	1368	1359	
Age (years)	56.3 (14.5)	51.6 (13.3)	48.3 (12.4)	<0.001
BMI (kg/m^2^)	22.8 (3.3)	22.8 (3.3)	22.8 (3.3)	0.96
Total cholesterol (mg/dL)	201 (39)	208 (38)	213 (38)	<0.001

Total energy intake (kcal/day)	1841 (383)	1868 (364)	1892 (344)	<0.001
Protein (g/day)	72.7 (16.3)	74.3 (16.2)	75.0 (15.0)	<0.001
Animal (g/day)	36.4 (11.5)	37.9 (12.0)	38.5 (11.3)	<0.001
Vegetable (g/day)	36.3 (7.9)	36.4 (7.6)	36.6 (7.1)	0.57
Protein (g/1000 kcal)	39.6 (4.9)	39.9 (4.9)	39.8 (5.0)	0.30
Animal (g/1000 kcal)	19.8 (5.0)	20.3 (5.0)	20.4 (5.0)	0.003
Vegetable (g/1000 kcal)	19.9 (2.5)	19.6 (2.6)	19.5 (2.4)	<0.001

Current alcohol drinker (%)	4.8	7.9	7.8	<0.001
Current cigarette smoker (%)	9.4	8.0	10.7	0.05

Averages are shown except for current alcohol drinker and cigarette smoker.

Parentheisis means standard deviation.

BMI means body mass index.

The association between serum albumin levels and animal or vegetable protein intake was estimated by multiple linear regression models (Table 4). In model 1 and model 2, animal protein was positively associated with serum albumin levels, although the coefficients of animal protein were very small. On the other hand, there is no clear association observed between vegetable protein and serum albumin levels. Regardless of sex and models, age was inversely associated with serum albumin levels with statistically significance. Standardized coefficients of age were considerably larger in both sexes than other variables. BMI was positively associated with serum albumin levels in model 2, but adjustment for BMI in model 2 hardly altered the coefficients of animal or vegetable protein intake, compared to those in model 1. Adding current cigarette smoking and current alcohol drinking to model 1, or entering animal or vegetable protein intakes per 1000 kcal into the model 2, instead of animal or vegetable protein intakes, did not change the results of animal or vegetable protein intake so much. Adjustment for total cholesterol in model 3 attenuated the association between animal protein intake and serum albumin level. Any of adjusted coefficients of determination in these models were relatively small, especially in women.

**Table 4.** Association beween serum albumin levels and protein intakes (animal, vegetable) with multiple linear regression by sex, 1990, Japan

	Men			
	
(Dependent variables)	Independent variables: serum albumin (g/L)			
	
Model 1	Coefficient	95% CI	Sandardized coefficient	*P* value
	
Age (10 years)	−1.18	−1.24 to −1.11	−0.52	<0.001
Animal protein (1SD, 14.5 g/day)	0.11	0.01 to 0.20	0.04	0.03
Vegetable protein (1SD, 9.2 g/day)	−0.06	−0.16 to 0.03	−0.02	0.19
	Adjusted coefficient of determination (*R*^2^) = 0.28			
	
Model 2	coefficient	95% CI	Sandardized coefficient	*P* value
	
Age (10 years)	−1.15	−1.22 to −1.08	−0.51	<0.001
BMI (1SD, 3.0 kg/m^2^)	0.34	0.25 to 0.43	0.11	<0.001
Animal protein (1SD, 14.5 g/day)	0.09	−0.01 to 0.18	0.03	0.08
Vegetable protein (1SD, 9.2 g/day)	−0.09	−0.18 to 0.00	−0.03	0.06
	Adjusted coefficient of determination (*R*^2^) = 0.29			
	
Model 3	coefficient	95% CI	Sandardized coefficient	*P* value
	
Age (10 years)	−1.14	−1.20 to −1.07	−0.51	<0.001
BMI (1SD, 3.0 kg/m^2^)	0.18	0.09 to 0.27	0.06	<0.001
Total cholesterol (1SD, 36.8 mg/dL)	0.61	0.52 to 0.70	0.20	<0.001
Animal protein (1SD, 14.5 g/day)	0.03	−0.07 to 0.12	0.01	0.55
Vegetable protein (1SD, 9.2 g/day)	−0.06	−0.15 to 0.03	−0.02	0.21
	Adjusted coefficient of determination (*R*^2^) = 0.33			
	

	Women			
	
(Dependent variables)	Independent variables: serum albumin (g/L)			
	
Model 1	coefficient	95% CI	Sandardized coefficient	*P* value
	
Age (10 years)	−0.47	−0.52 to −0.42	−0.25	<0.001
Animal protein (1SD, 11.6 g/day)	0.10	0.02 to 0.18	0.04	0.02
Vegetable protein (1SD, 7.6 g/day)	0.03	−0.05 to 0.11	0.01	0.50
	Adjusted coefficient of determination (*R*^2^) = 0.07			
	
Model 2	coefficient	95% CI	Sandardized coefficient	*P* value
	
Age (10 years)	−0.48	−0.54 to −0.43	−0.26	<0.001
BMI (1SD, 3.3 kg/m^2^)	0.14	0.06 to 0.22	0.05	<0.001
Animal protein (1SD, 11.6 g/day)	0.09	0.01 to 0.17	0.04	0.02
Vegetable protein (1SD, 7.6 g/day)	0.02	−0.06 to 0.10	0.01	0.66
	Adjusted coefficient of determination (*R*^2^) = 0.07			
	
Model 3	coefficient	95% CI	Sandardized coefficient	*P* value
	
Age (10 years)	−0.62	−0.68 to −0.56	−0.33	<0.001
BMI (1SD, 3.3 kg/m^2^)	0.03	−0.05 to 0.10	0.01	0.44
Total cholesterol (1SD, 38.8 mg/dL)	0.65	0.58 to 0.73	0.25	<0.001
Animal protein (1SD, 11.6 g/day)	0.04	−0.04 to 0.12	0.02	0.34
Vegetable protein (1SD, 7.6 g/day)	0.03	−0.05 to 0.11	0.01	0.41
	Adjusted coefficient of determination (*R*^2^) = 0.12			

CI means confidence interval. BMI means body mass index.

## DISCUSSION

In the present study, very weak positive association between animal protein and serum albumin level was observed. On the other hand, substantially strong relation between age and serum albumin level was also observed. Compared to age, associations of animal protein intake or BMI were very small, and coefficients of animal protein after adjustment for age were also considerably small. Accordingly, association between animal protein and serum albumin level might be essentially small even if it is statistically significant.

Adjustment for BMI did not change the results so much, and coefficients of BMI were also small. Although BMI was considered as one of markers of nutrition status, present results suggested BMI did not work as a strong confounding factor in the association between animal protein and serum albumin level. There are few individuals with extreme malnutrition or very low BMI in Japan recently; and the range of BMI are relatively narrow as a whole. Accordingly, BMI might not be a marker of nutrition status at least in the general population.

A few studies reported the interaction between cholesterol and albumin level in the relationship between serum albumin levels and mortality, disease incidences or activities of daily living,^14^^–^^16^ although the interaction is also still controversial. In present analyses, adjustment for total cholesterol considerably attenuated the association between animal protein intake and serum albumin level, and the association of total cholesterol with albumin was strong next to age. Cholesterol is synthesized mainly in the liver as well as albumin, and both albumin and cholesterol reflects liver synthesis function. Accordingly relatively strong relation between them is reasonable. Adjustment for total cholesterol means adjusting liver synthesis function, so the attenuation of the association between animal protein intakes and serum albumin levels in model 3 might be reasonable because protein intake is also a part of albumin synthesis pathway in the liver.

The present study had several limitations. First, some prospective studies reported the possibility of existence of the threshold of low albuminemia in relation with adverse health status.^3^^–^^6^^,^^14^^–^^16^ However, since prevalence of low albuminemia, such as albumin level of 38 g/L or less in the present analyses was very low, it was difficult to investigate the threshold of serum albumin levels. Second, detailed medical histories of each subject that might influence on serum albumin levels or protein intakes were unknown, such as nephrotic syndrome or liver cirrhosis. Third, the nutritional data of each household member is indirectly estimated on the basis of other reference data, so the nutritional data might not accurately reflect the individual nutrition intake. The possibility of unknown influence of this method on the present results could not be completely excluded.

In conclusion, present analysis indicated the possibility that animal protein intake was related with serum albumin levels, while vegetable protein intake was not related. Further investigation was needed to estimate the association between protein intakes and serum albumin levels, considering the above mentioned limitations.

## References

[1] Phillips A , Shaper AG , Whincup PH Association between serum albumin and mortality from cardiovascular disease, cancer, and other causes . Lancet. 1989;2:1434–6 10.1016/S0140-6736(89)92042-4 2574367

[2] Gillum RF , Makuc DM Serum albumin, coronary heart disease, and death . Am Heart J. 1992;123:507–13 10.1016/0002-8703(92)90667-K 1736588

[3] Corti MC , Guralnik JM , Salive ME , Sorkin JD Serum albumin level and physical disability as predictors of mortality in older persons . JAMA. 1994;272:1036–42 10.1001/jama.272.13.1036 8089886

[4] Fried LP , Kronmal RA , Newman AB , Bild DE , Mittelmark MB , Polak JF , Risk factors for 5-year mortality in older adults: the Cardiovascular Health Study . JAMA. 1998;279:585–92 10.1001/jama.279.8.585 9486752

[5] Djoussé L , Rothman KJ , Cupples LA , Levy D , Ellison RC Serum albumin and risk of myocardial infarction and all-cause mortality in the Framingham Offspring Study . Circulation. 2002;106:2919–24 10.1161/01.CIR.0000042673.07632.76 12460872

[6] Okamura T , Hayakawa T , Kadowaki T , Kita Y , Okayama A , Elliott P , A combination of serum low albumin and above-average cholesterol level was associated with excess mortality . J Clin Epidemiol. 2004;57:1188–95 10.1016/j.jclinepi.2004.02.019 15612140

[7] Schalk BW , Visser M , Bremmer MA , Penninx BW , Bouter LM , Deeg DJ Change of serum albumin and risk of cardiovascular disease and all-cause mortality: Longitudinal Aging Study Amsterdam . Am J Epidemiol. 2006;164:969–77 10.1093/aje/kwj312 16980573

[8] Ministry of Health and Welfare. The National Survey on Circulatory Disorders, 1990. Tokyo: Japan Heart Foundation; 1992 (in Japanese).

[9] Ministry of Health and Welfare. The National Nutrition Survey in Japan, 1990. Tokyo: Daiichi Shuppan; 1992 (in Japanese).

[10] Okamura T , Hayakawa T , Kadowaki T , Kita Y , Okayama A , Ueshima H; NIPPON DATA90 Research Group The inverse relationship between serum high-density lipoprotein cholesterol level and all-cause mortality in a 9.6-year follow-up study in the Japanese general population . Atherosclerosis. 2006;184:143–50 10.1016/j.atherosclerosis.2005.03.042 15913635

[11] Hozawa A , Okamura T , Kadowaki T , Murakami Y , Nakamura K , Hayakawa T , Gamma-glutamytransferase predicts cardiovascular death among Japanese women . Atherosclerosis. 2007;194:498–504 10.1016/j.atherosclerosis.2006.08.058 17034795

[12] Kadota A , Hozawa A , Okamura T , Kadowak T , Nakmaura K , Murakami Y , Relationship between metabolic risk factor clustering and cardiovascular mortality stratified by high blood glucose and obesity: NIPPON DATA90, 1990–2000 . Diabetes Care. 2007;30:1533–8 10.2337/dc06-2074 17363755

[13] Okuda N , Miura K , Yoshita K , Matsumura Y , Nakamura Y , Okayama A , Integration of data from NIPPON DATA80/90 and National Nutrition Survey in Japan: for cohort studies of representative Japanese on nutrition . J Epidemiol. 2010;20 Suppl 3;S506–14 10.2188/jea.JE20090218 20351471 PMC3920387

[14] Weijenberg MP , Feskens EJ , Souverijn JH , Kromhout D Serum albumin, coronary heart disease risk, and mortality in an elderly cohort . Epidemiology. 1997;8:87–92 10.1097/00001648-199701000-00014 9116102

[15] Okamura T , Hayakawa T , Hozawa A , Kadowaki T , Murakami Y , Kita Y , Lower levels of serum albumin and total cholesterol associated with decline in activities of daily living and excess mortality in a 12-year cohort study of elderly Japanese . J Am Geriatr Soc. 2008;56:529–35 10.1111/j.1532-5415.2007.01549.x 18179493

[16] Reuben DB , Ix JH , Greendale GA , Seeman TE The predictive value of combined hypoalbuminemia and hypocholesterolemia in high functioning community-dwelling older persons: MacArthur Studies of Successful Aging . J Am Geriatr Soc. 1999;47:402–6 10203113 10.1111/j.1532-5415.1999.tb07230.x

